# Complex interventional treatment in a patient with atrial fibrillation and stroke caused by large carotid artery thrombus: a case report

**DOI:** 10.1186/s12883-015-0322-4

**Published:** 2015-04-24

**Authors:** Anetta Lasek-Bal, Tomasz Urbanek, Damian Ziaja, Aldona Warsz-Wianecka, Przemysław Puz, Krzysztof Ziaja

**Affiliations:** Department of Neurology, Medical University of Silesia Hospital No. 7, Professor Leszek Giec Upper Silesian Medical Centre, Katowice, Poland; School of Health Science, Medical University of Silesia, Katowice, Poland; Department of General Surgery and Angiology, Medical University of Silesia Hospital No. 7, Professor Leszek Giec Upper Silesian Medical Centre, Katowice, Poland

**Keywords:** Stroke, Atrial fibrillation, Arterial thrombolysis, Embolectomy, Thrombectomy

## Abstract

**Background:**

The treatment option for acute ischaemic stroke depends on the duration of symptoms, the dynamics of neurological condition changes, the aetiology, type of stroke, as well as the results of angiographic and neuroimaging tests.

**Case presentation:**

A 60-year-old male patient presented with progressive left hemisphere stroke caused by extensive cardiogenic embolism of the common carotid artery and a thrombus closing the internal carotid artery from its ostium to the level of its intracranial division. The complex revascularisation therapy involving surgical embolectomy of the common carotid artery, thrombectomy of the internal carotid artery and intra-arterial thrombolysis has led to the improvement of arterial patency and has countered the progression of acute cerebral ischaemia.

**Conclusion:**

Emergency carotid embolectomy together with thrombectomy and local thrombolytic rt-PA treatment may be a reasonable rescue therapy for carefully selected patients with large-vessel acute stroke. Further research is needed to establish the advantages and safety of surgical thrombectomy in patients with acute embolic occlusion of the carotid artery and ineffectiveness of or contraindications for systemic thrombolytic treatment.

## Background

Modern methods of treating acute cerebral ischaemia include interventional treatment such as cerebral vascular thrombectomy or local thrombolytic therapy. The application of thrombectomy and loco-regional thrombolytic treatment is recommended for a select cohort of patients who neither qualify for, nor benefit from, intravenous thrombolysis [1]. When planning this type of treatment, one must consider both the general condition, the degree of neurological dysfunction of the patient and the presence of potential contraindications, especially those related to thrombolytic treatment. Significant factors determining the success of the procedure include the duration of the ischaemia, the location (accessibility) and extent of lesions, and also the technical capabilities of achieving patency of the vascular tree. Where patients experience acute ischaemia in the cerebral circulation due to embolic complications, one should especially focus on the cardiogenic background of the embolism. Another reason for embolism may arise from material moving from unstable plaques. Because of the size of embolic material, the majority of such lesions are located in intracranial vessels or in the internal carotid artery, especially in its distal segments or in its intracranial branches. In the case of large-volume embolic material, occlusion tends to affect vessels in the proximal section, e.g., in the common carotid artery bulb. The presence of such extensive lesions, most often with thrombosis of the arteries above, as well as the embolic nature of the occlusion may constitute a significant problem for performing a quick and effective intravascular procedure to restore patency.

## Case presentation

A 60-year-old Caucasian male patient was admitted to the Neurology Department at the Academic Medical Centre because of speech impairment and right limb weakness. Onset was sudden and symptoms had already been present for approximately 4 hours. The patient’s medical history revealed long-standing arterial hypertension, ischaemic heart disease and nicotinism.

**Figure 1:**
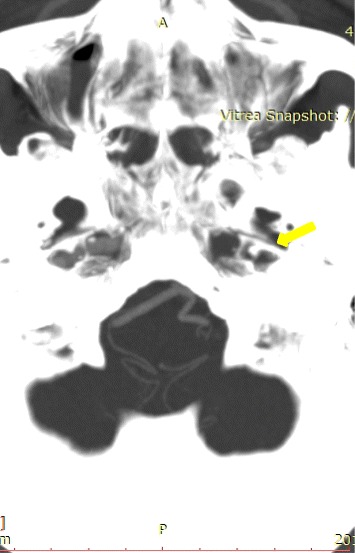
Thrombus in the left internal carotid artery (cavernous part).

**Figure 2:**
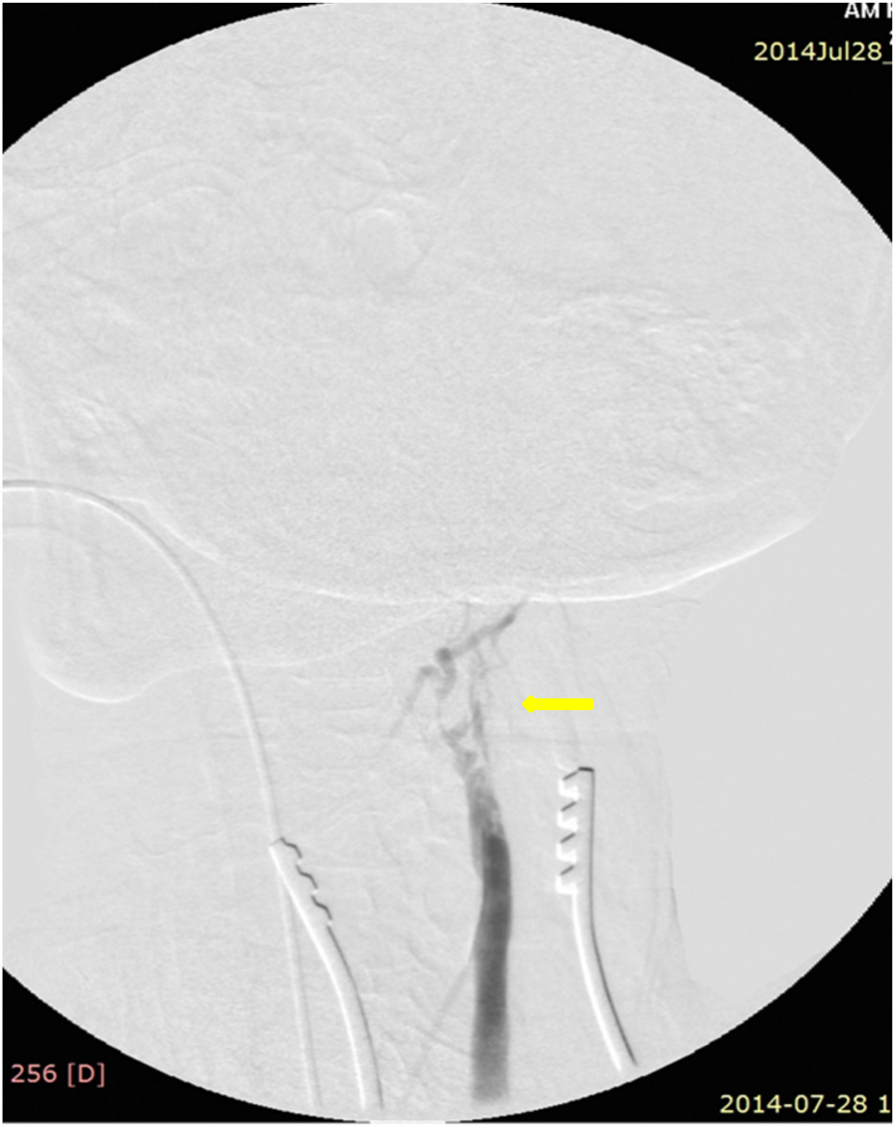
Occlusion of left internal carotid artery.

**Figure 3:**
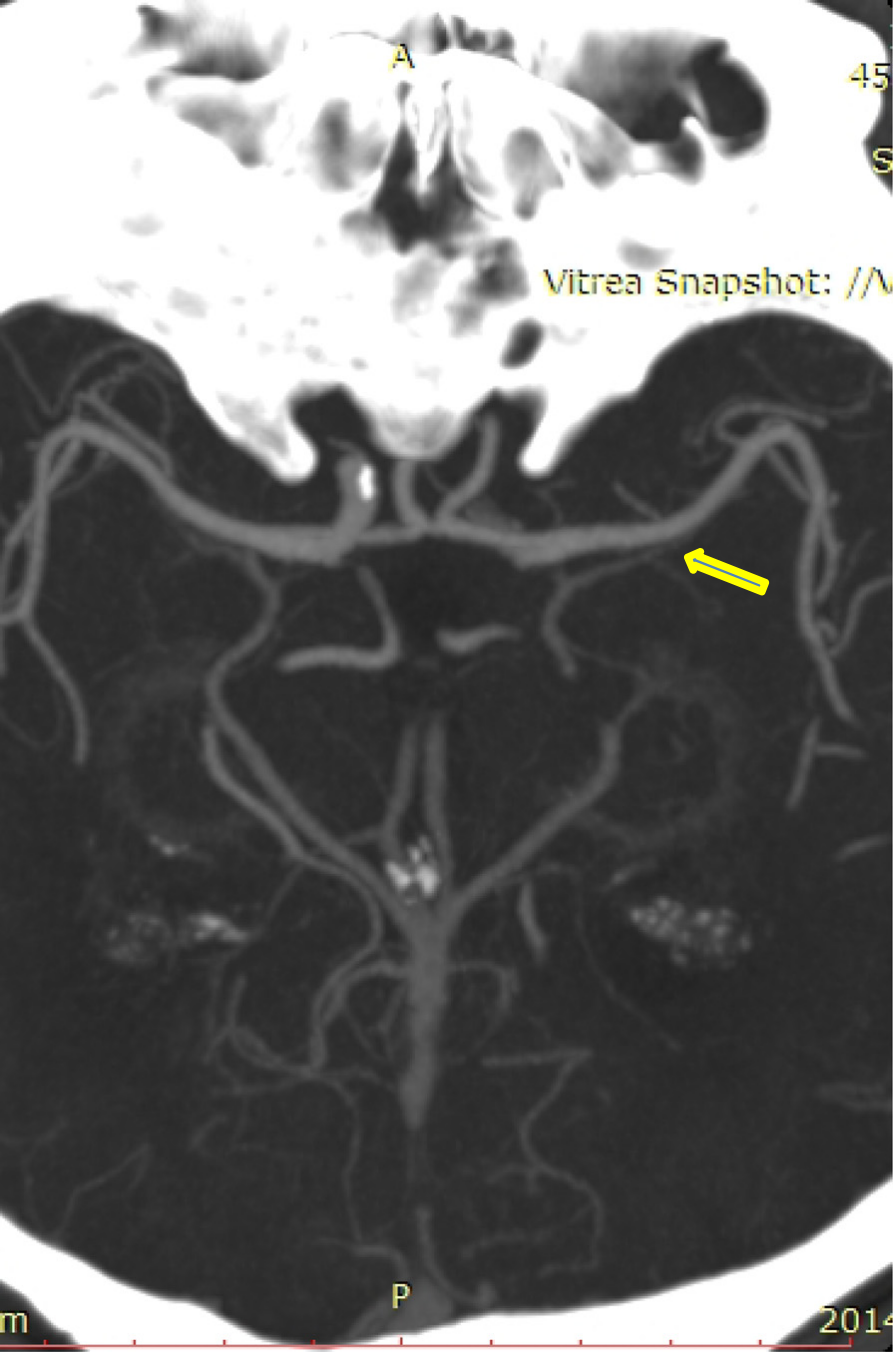
Preserved blood flow in left side intracranial arteries.

An examination performed after hospital admission revealed right progressive CNVII paresis, mixed aphasia and paresis of right limbs [2/5 in Medical Research Council Muscle strength scale]. Computed tomography (CT) of the head revealed an acute ischaemic focus in the left hemisphere and white matter lesions, a surrogate for cerebral small-vessel disease. The electrocardiogram recorded atrial fibrillation with a ventricular rate of 70 beats/min. Ultrasound examination and angio-CT scan (arterial and venous phase) of the head revealed a left-sided occlusion of the common carotid artery (CCA), external carotid artery, and the internal carotid artery (ICA) at the cavernous section (with an extended thrombus in the ICA above the embolism) (Figures 1 and 2). At this time, preserved blood flow was confirmed in the middle cerebral artery (MCA) and anterior cerebral artery (ACA) on the left side (Figure 3). In the ultrasound duplex Doppler examination, the material occluding the CCA was hyperechogenic, with thrombus extension into the ICA, suggesting the presence of fibrous embolic material migration into the CCA, occlusion of the ICA and propagation of the thrombosis into the distal part of the carotid artery. The movement of the embolism in the CCA was visible during the ultrasound examination with distal CCA bulb occlusion and no signs of dissection or aneurysm.

Because of the duration of the symptoms, the unstable neurological condition of the patient, the extent and nature of lesions occluding the arteries, and the newly created cerebral ischaemia focus, a decision was made to undertake combined interventional treatment involving surgical and endovascular therapy. Because of the size and nature of the embolic material (mostly hyperechogenic, probably fibrous and non-compressible lesion), surgical embolectomy was undertaken in the first step of the procedure with the endovascular methods reserved for adjunct treatment if required.

**Figure 4:**
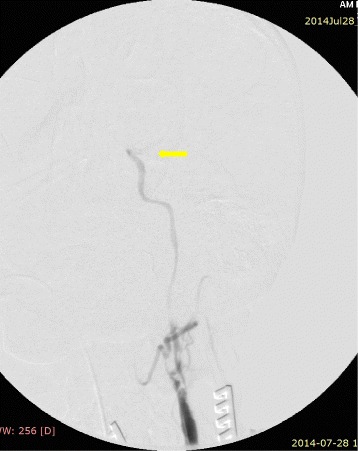
Occlusion in region of internal carotid artery bifurcation.

**Figure 5:**
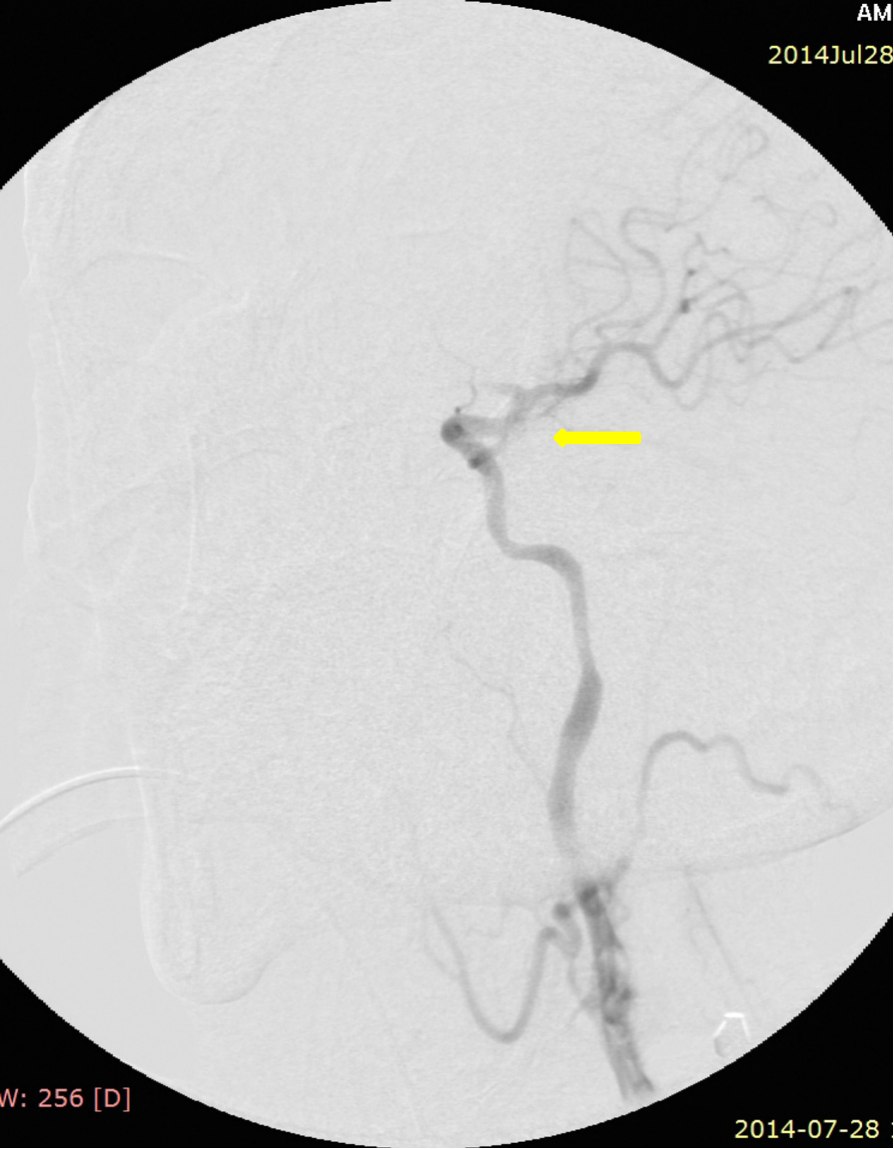
Restored flow in internal carotid artery and anterior cerebral artery.

The procedure was performed in a hybrid operating room under fluoroscopic control (started in the 6th hour from the start of symptoms). From the incision on the front edge of the left sternocleidomastoid muscle, the ICA and its division were dissected. A transverse cut on the CCA was made, just below the ostium of the ICA. Through arteriotomy, an extensive embolism of the CCA bulb was removed: 10 × 7 mm in size, hard, with fibrous structure. After removing the embolism, a good inflow and lack of back flow were recorded. Using a 3 F Fogarty catheter, a 10-cm thrombus was removed from the ICA; after removing the embolus, arteriography showed restored flow in the ICA, but lack of contrast in the cerebral circulation corresponding to the ACA and MCA (Figure 4). Based on the arteriographic image, a decision was made to implement thrombolytic treatment: 0.3 mg/kg of recombinant tissue plasminogen activator (rt-PA; Actylise, Boehringer Ingelheim, Germany) was administered through a catheter placed in the ICA. Follow-up angiography revealed blood flow in the ICA and the ACA (Figure 5). The arteriotomy was sutured using a vascular suture (Prolene 5–0). Because of parenchymatous bleeding from the wound caused by administering rt-PA and heparin (2500 U; as a routine surgical measure), haemostatic dressing and suturing were used, while closure was delayed until the next day to allow normalisation and prevent clotting abnormalities.

**Figure 6:**
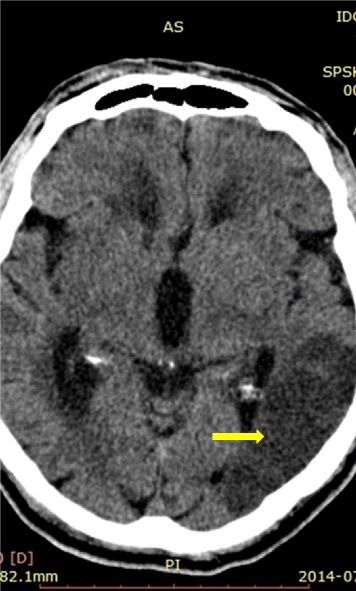
Ischaemic focus in left brain hemisphere.

Systematic improvement of the neurological condition continued in the post-surgery period without complications. During anticoagulant therapy, unfractionated heparin was used at an initial daily dose of 15000 U/24 h, which was later changed to 25000 U/24 h based on the activated partial thromboplastin time. Through continuous heart rate monitoring, atrial fibrillation was observed. Follow-up ultrasound and angio-CT of the head (1st day following the intervention) revealed proper blood flow in the affected arteries, but a CT scan of the head revealed a marked ischaemic focus (Figure 6). On the 10th day from the onset of the symptoms, the patient left the ward in a neurologically good condition, with discrete aphasia, yet able to move independently. Based on the clinical course and results of additional tests (imaging and electrophysiological testing), it was determined that the stroke occurred in the patient as a result of cardiogenic embolism from atrial fibrillation (diagnosed de novo during hospitalisation). As a further prophylaxis against stroke, dabigatran in daily dose of 300 mg was introduced from the 10th day. The written informed consent of the patient was obtained for participation in this study and for publication of the results.

## Discussion

The treatment option for acute ischaemic stroke depends on the duration of symptoms, the dynamics of any neurological condition changes, the aetiology of the stroke, as well as the results of angiographic and neuroimaging tests. As per current recommendations, initial considerations include reperfusion methods based on intravenous thrombolysis or, in selected cases, intra-arterial thrombolysis. Whether greater benefits are obtained from applying mechanical thrombectomy as the only form of treatment or in combination with pharmacological thrombolysis is currently the subject of research. According to updated guidelines, thrombectomy may be performed in patients with cerebral stroke induced by acute critical narrowing or occlusion of intracranial arteries, which neither qualify for, nor benefit from, intravenous thrombolysis [1]. Cerebral stroke due to acute cerebral and/or carotid artery occlusion is characterised by severe progression and poor outcome [2]. In such cases, complete arterial recanalisation after intravenous rt-PA administration is observed in as few as 10–11% of patients [3-6]. In the “carotid T-occlusion” (thrombolytic occlusion from the proximal ICA section to the ACA and MCA), the prognosis is uncertain, even if recanalisation is performed within a few hours. Brekenfeld et al. [7] report a favourable outcome in only 17% of patients in the 3rd month post-stroke (mRankin ≤ 2) despite observed ICA, MCA and ACA recanalisation in 63%, 17% and 33% of cases, respectively [7].

The extent of cerebral ischemia depends on the compensatory capabilities of collateral circulation in the Circle of Willis or leptomeningeal anastomosis, and on the capability to restore brain circulation within the therapeutic window. Endovascular interventions may be more effective than intravenous and/or intra-arterial thrombolysis [8]. Although there is no doubt that, when applied within a short time from the onset of the disease, endovascular approaches result in a more favourable prognosis, these approaches may still be implemented during the extended therapeutic window, i.e., from 8 to 12 h from the onset of the stroke [9-11]. When considering that cerebral stroke patients are often admitted to hospital beyond the thrombolytic therapeutic window, endovascular therapy is a favourable alternative for the majority of occluded arteries.

Emergency surgical embolectomy in patients with carotid artery occlusion in the acute stroke phase is rarely considered. Yet, it is worth noting that 10–20% of all strokes within the anterior part of the Circle of Willis are associated with ICA pathology [12]. In such cases, the occluded downstream intracranial vessels (e.g., MCA) may be recanalised by systemic or local thrombolysis. Unblocking intracranial vessels using a thrombolytic drug despite residual occlusion of the ICA may only result in temporary reperfusion. Achieving permanent improvement of cerebral artery patency depends on the condition of the carotid artery that feeds it. This is true especially in cases with T-occlusions, which is exemplified in the present patient. The dynamics of intravascular lesions in our patient may be tracked based on radiographic examinations. Within 2 h of the angio-CT examination upon admission to hospital and the performed procedure, an expansion of the occlusion from the level of the ICA cavernous section to the ACA and MCA occurred. Only a comprehensive reperfusion strategy, such as a CCA embolectomy with ICA thrombectomy and local thrombolysis, provided an opportunity for quick restoration of vessel patency and for preventing further progression of cerebral ischaemia, such as found here. The decision to select a mixed revascularisation treatment was based on the understanding that the effectiveness of thrombolytic treatment in the case of “old” fibrous embolic lesions is limited and that their size significantly limits the possibility of complete embolism removal. Observations to date with the use of carotid artery embolectomy in treating strokes are scarce, and their results are variable. The interpretation of those results is all the more difficult as reports involve both patients with cardiogenic carotid artery emboli and patients with an occlusion of the ICA resulting from atherosclerotic thrombosis with intracranial progression [12-14]. However, some reports describe the positive effect of thrombectomy and surgical embolectomy in patients previously not subjected to thrombolytic therapy, or where thrombolytic therapy was insufficient [12,15,16].

Perhaps because of diagnostic difficulties and the necessary time window, descriptions of surgical embolectomy of the ICA and its branches are still rare. Touho et al. [4] presented results where the CCA was effectively unblocked with the aid of surgical embolectomy in four patients, which constituted 66.7% of performed interventions. Murata et al. [17] describes three cases of effective surgical embolectomy of the common and internal carotid arteries. Finally, Inoue et al. [18] reveals positive outcomes from embolectomy following a retrospective analysis of 23 patients with occlusion of the ICA and/or its branches. There is no consensus at present on the possibility and efficacy of endovascular treatment in patients with acute ischaemic stroke due to atherosclerotic occlusion of the extracranial ICA. This type of stroke, caused by acute occlusion of the ICA, is usually associated with significant morbidity and mortality. In the study published by Papanagiotou et al. [19], the authors demonstrated a high technical success rate of carotid artery stenting in acute extracranial ICA occlusion. In their paper, the clinical outcomes of the extracranial revascularisation were evaluated in a group of 22 patients; in 18 patients, an additional intracranial occlusion of ICA or MCA was found and treated with endovascular methods. Successful recanalisation of extracranial ICA was achieved in 95% of cases and intracranial vessel recanalisation was obtained in 61% of cases with an overall recanalisation rate of 63%. During follow-up, nine of 22 patients (41%) had a modified Rankin Score of ≤2 at 90 days with observed mortality rate in the whole cohort of 13.6% at 90 days [19].

In the case described here, owing to the dynamics of clinical and radiological lesions, and the presence of a multi-level acute occlusion of both the CCA and ICA and its branches, a decision was made to proceed surgically, with subsequent intra-arterial rt-PA. The decision to undertake treatment resulted from the nature of the above-mentioned lesions. Very often, cardiogenic embolisms have the form of “hard” fibrous lesions, which considerably impacts the effectiveness of thrombolytic treatment. The effect of intravenous thrombolysis depends on the size of the embolism, while the opportunity for recanalisation of an artery with a cardiogenic embolism with a size of > 8 mm2 is slight [20]. However, because of the size and nature of the embolism (hard fibrous masses), the application of percutaneous mechanical thrombectomy was unlikely to succeed in complete removal of the embolism from the area of the CCA. Moreover, it could lead to fragmentation of the embolic material from manipulation of endovascular catheters with further transmission to smaller arteries and further cerebral ischaemia. The performance of a surgical procedure under fluoroscopic control, simultaneous application of intracranial treatment in the form of thrombectomy, and intra-arterial thrombolytic treatment made it possible to obtain quick recanalisation of treated vessels beyond the level of the primary closure.

## Conclusion

Emergency carotid embolectomy together with thrombectomy and local thrombolytic rt-PA treatment may be a reasonable rescue therapy for carefully selected patients with acute large-vessel stroke. Further trials are needed to establish the advantages and safety of surgical thrombectomy in patients with acute embolic occlusion of the carotid artery where systemic thrombolytic treatment is ineffective or contraindicated.

Written informed consent was obtained from the patient for publication of this Case report and any accompanying images.

## Abbreviations

CT: Computed tomography
CCA: Common carotid artery
ICA: Internal carotid artery
MCA: Middle cerebral artery
ACA: Anterior cerebral artery
rt-PA: Recombinant tissue plasminogen activator
